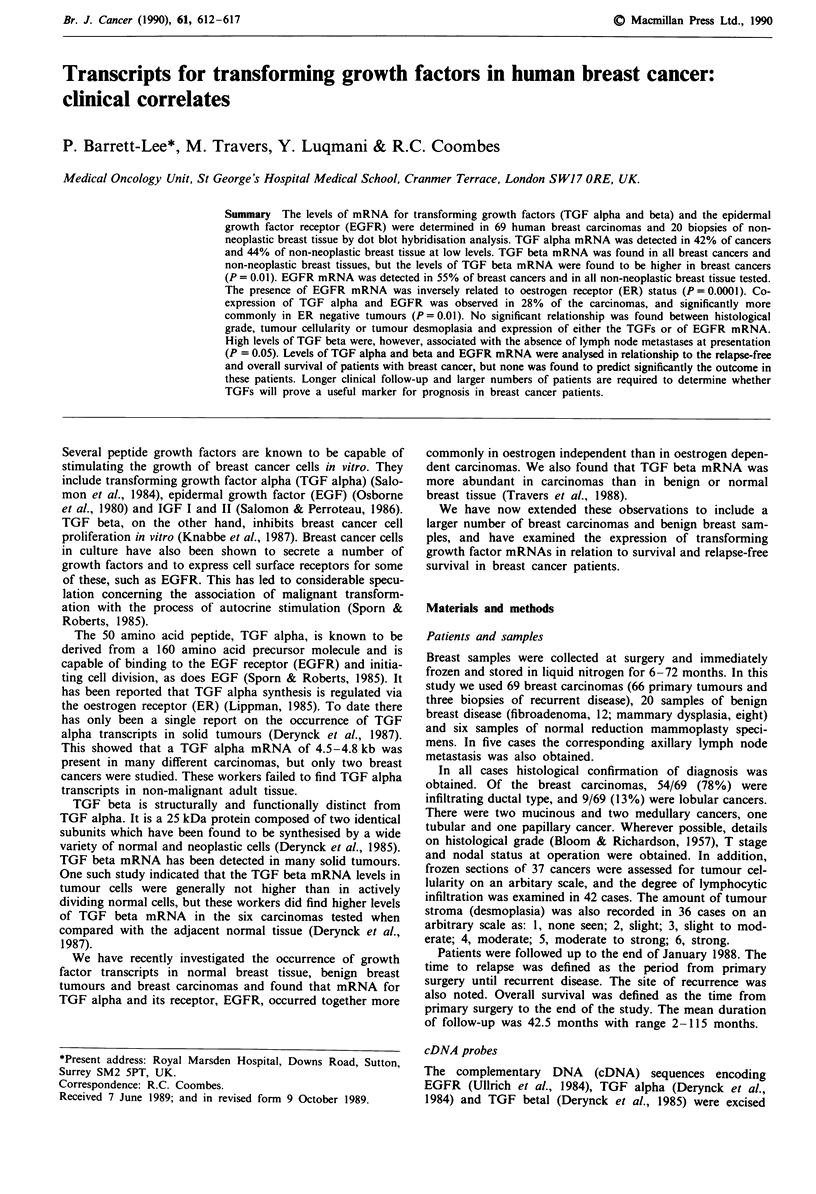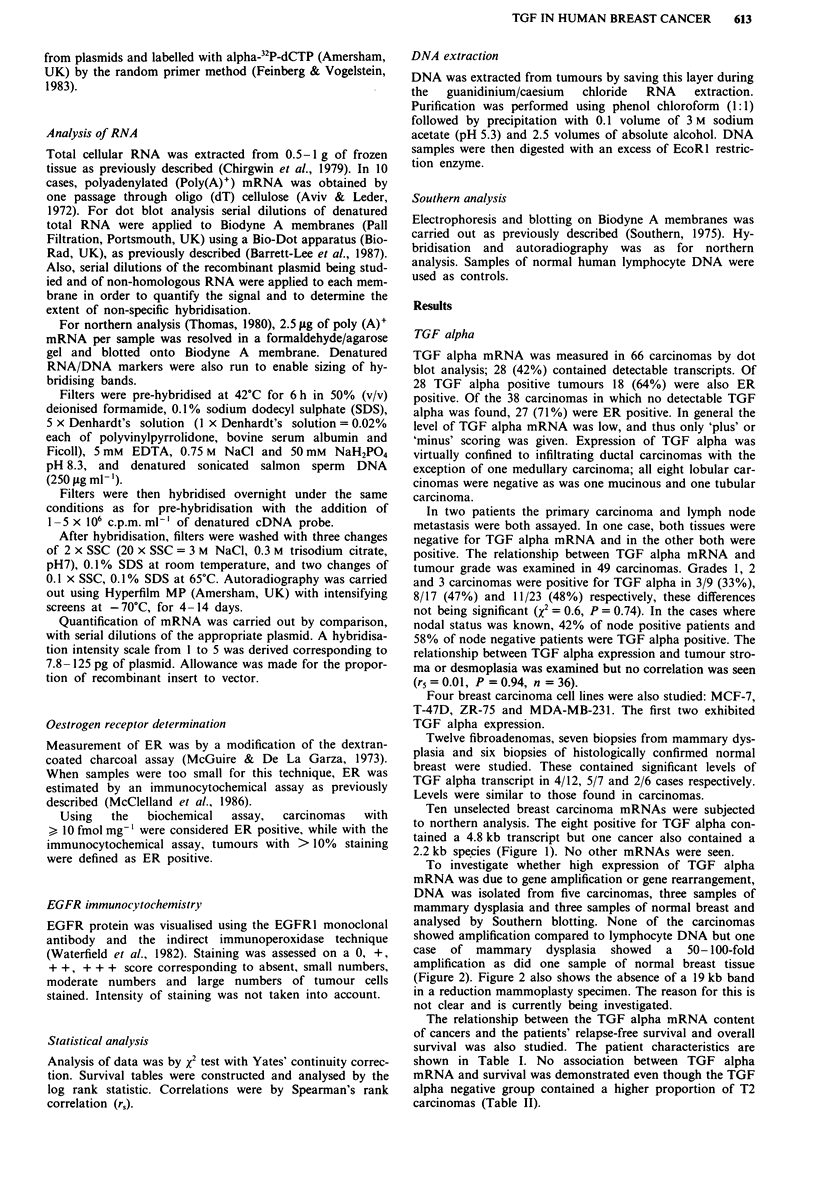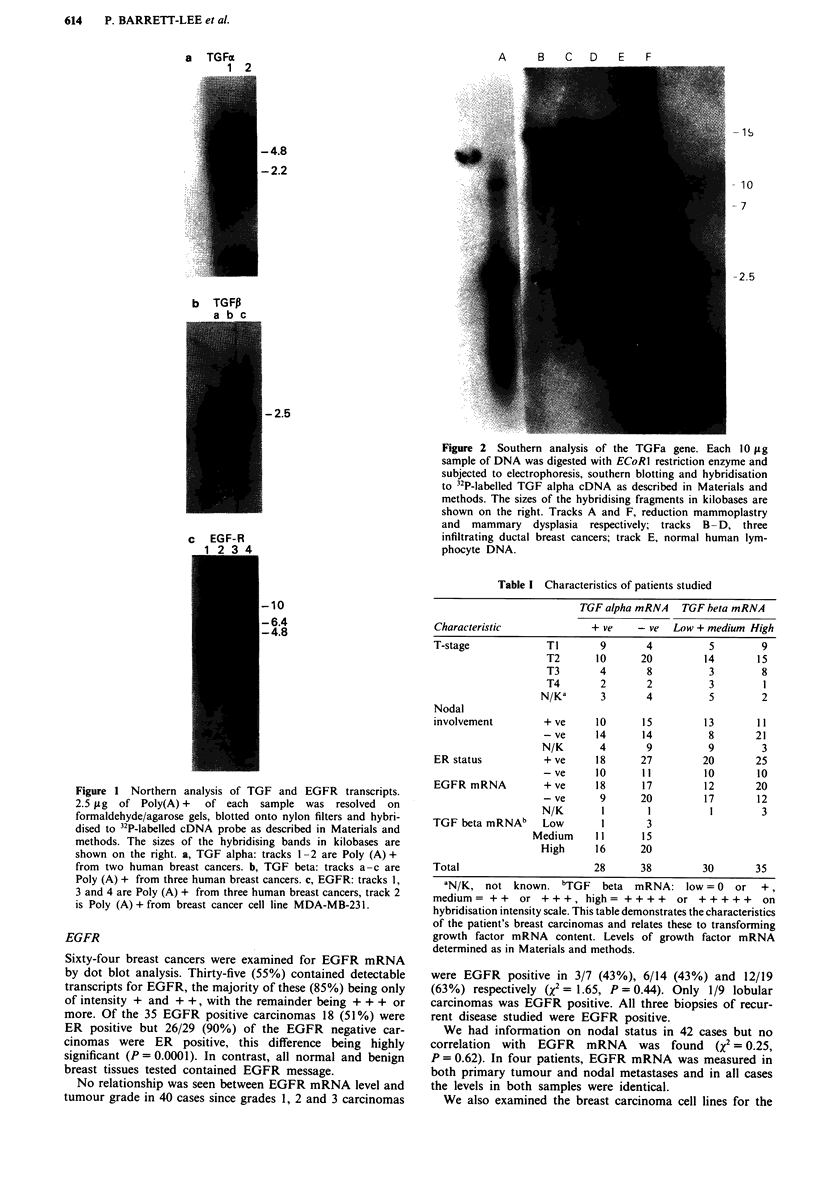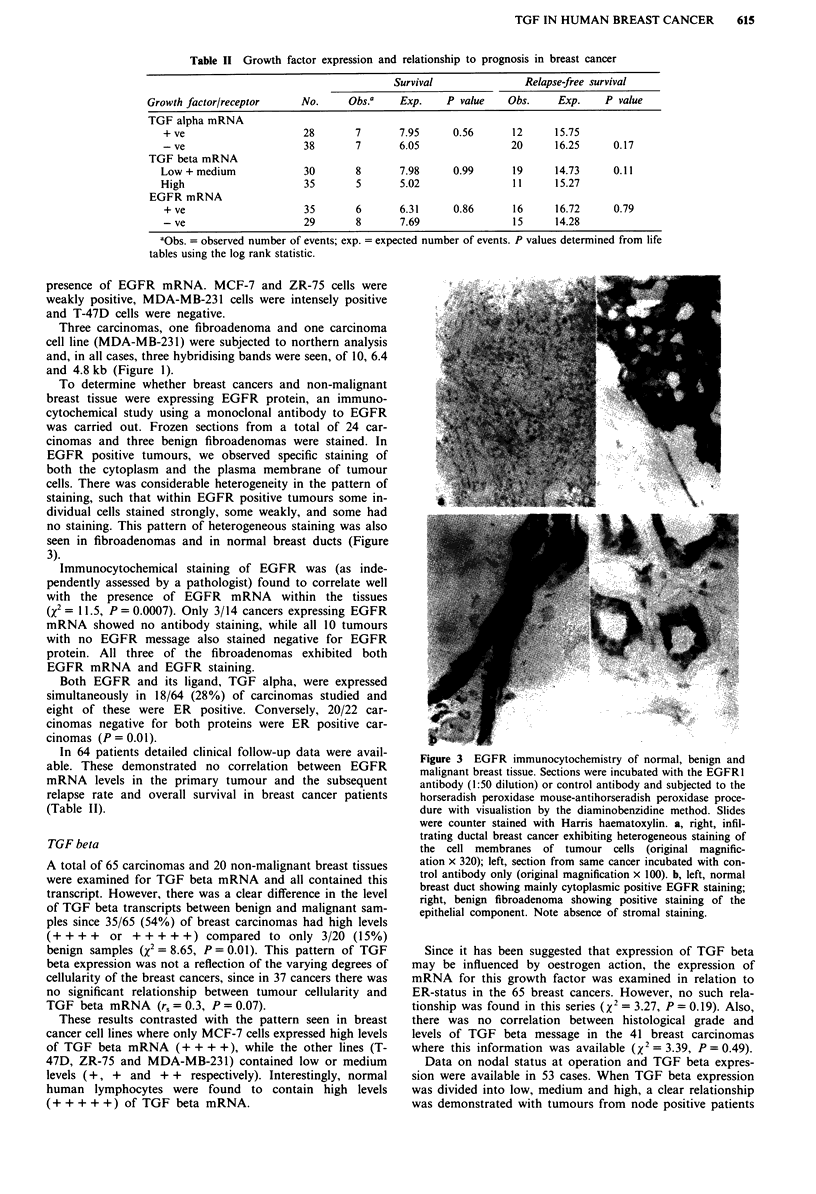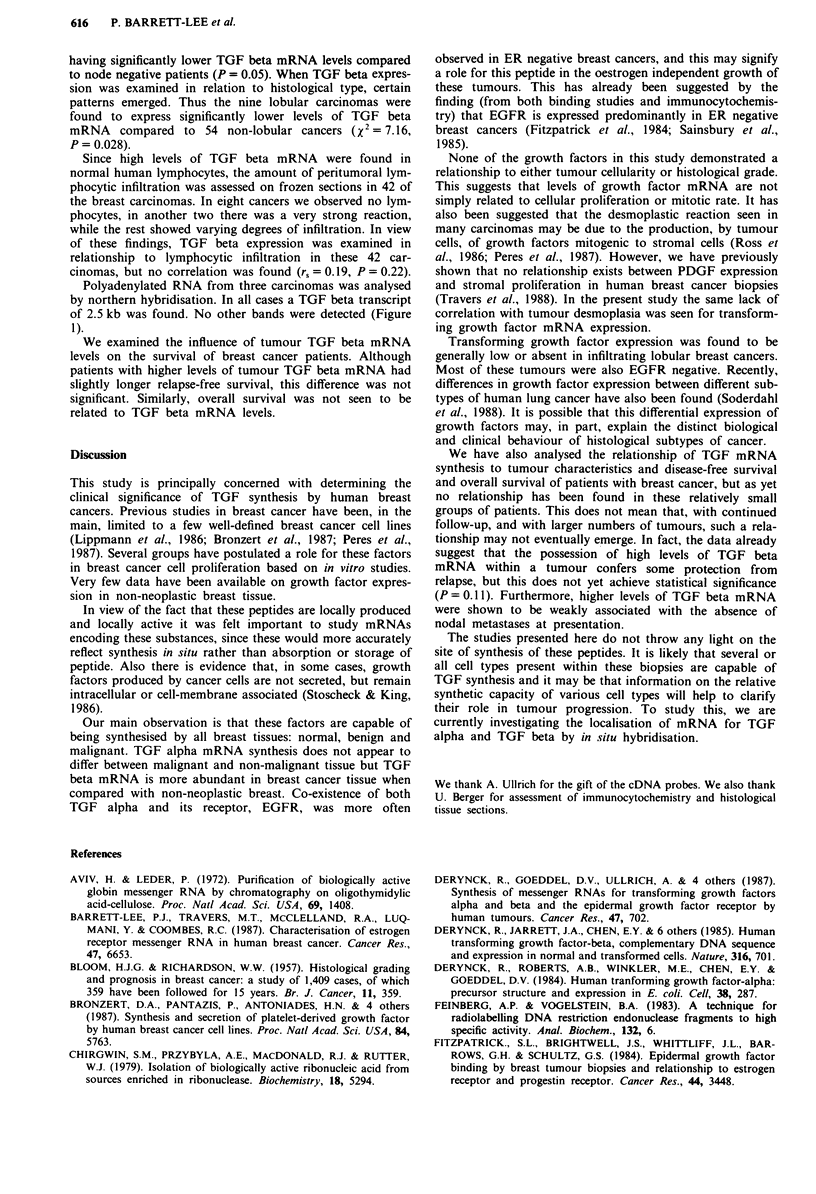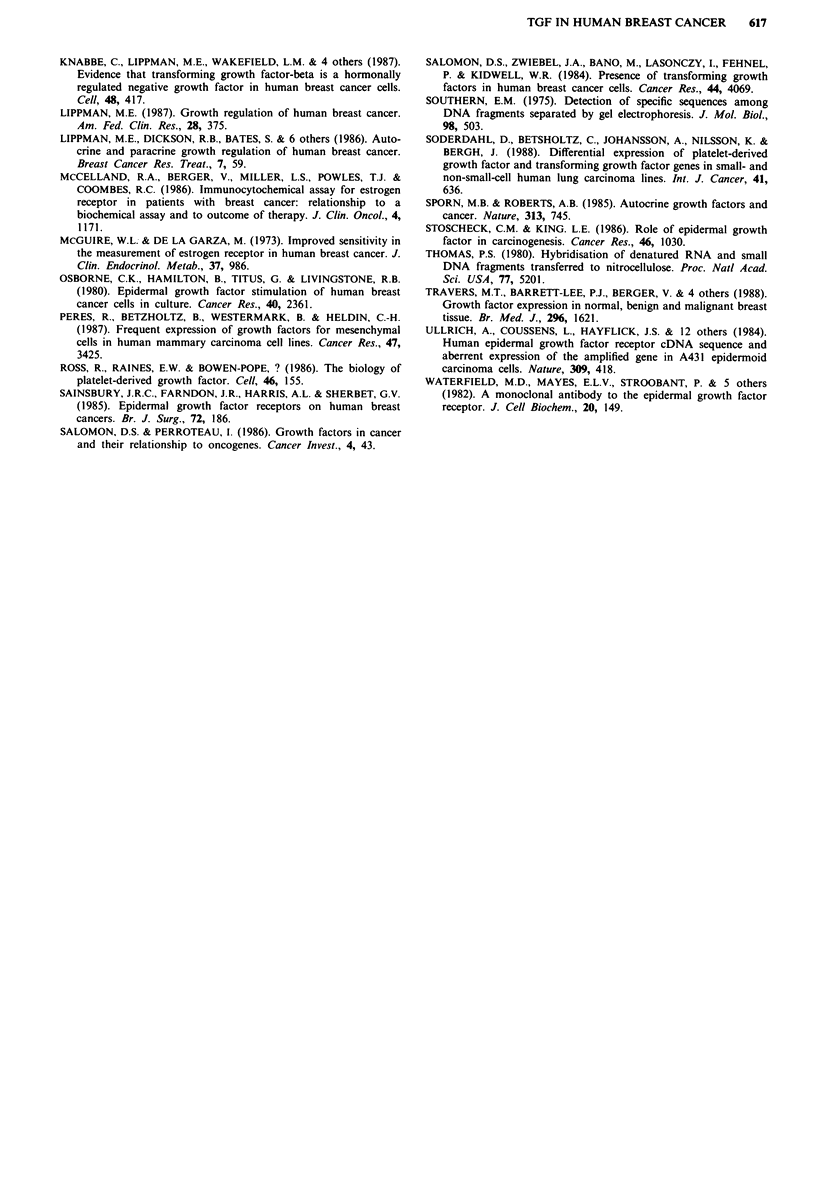# Transcripts for transforming growth factors in human breast cancer: clinical correlates.

**DOI:** 10.1038/bjc.1990.136

**Published:** 1990-04

**Authors:** P. Barrett-Lee, M. Travers, Y. Luqmani, R. C. Coombes

**Affiliations:** Medical Oncology Unit, St George's Hospital Medical School, London, UK.

## Abstract

**Images:**


Br. J. Cancer (1990), 61, 612-617 © Macmillan Press Ltd., 1990

### Transcripts for transforming growth factors in human breast cancer: clinical correlates

P. Barrett-Lee*, M. Travers, Y. Luqmani & R.C. Coombes

*Medical Oncology Unit, St George's Hospital Medical School, Cranmer Terrace, London SW17 0RE, UK.*

**Summary** The levels of mRNA for transforming growth factors (TGF alpha and beta) and the epidermal growth factor receptor (EGFR) were determined in 69 human breast carcinomas and 20 biopsies of non-neoplastic breast tissue by dot blot hybridisation analysis. TGF alpha mRNA was detected in 42% of cancers and 44% of non-neoplastic breast tissue at low levels. TGF beta mRNA was found in all breast cancers and non-neoplastic breast tissues, but the levels of TGF beta mRNA were found to be higher in breast cancers ($P = 0.01$). EGFR mRNA was detected in 55% of breast cancers and in all non-neoplastic breast tissue tested. The presence of EGFR mRNA was inversely related to oestrogen receptor (ER) status ($P = 0.0001$). Co-expression of TGF alpha and EGFR was observed in 28% of the carcinomas, and significantly more commonly in ER negative tumours ($P = 0.01$). No significant relationship was found between histological grade, tumour cellularity or tumour desmoplasia and expression of either the TGFs or of EGFR mRNA. High levels of TGF beta were, however, associated with the absence of lymph node metastases at presentation ($P = 0.05$). Levels of TGF alpha and beta and EGFR mRNA were analysed in relationship to the relapse-free and overall survival of patients with breast cancer, but none was found to predict significantly the outcome in these patients. Longer clinical follow-up and larger numbers of patients are required to determine whether TGFs will prove a useful marker for prognosis in breast cancer patients.

Several peptide growth factors are known to be capable of stimulating the growth of breast cancer cells *in vitro*. They include transforming growth factor alpha (TGF alpha) (Salomon *et al.*, 1984), epidermal growth factor (EGF) (Osborne *et al.*, 1980) and IGF I and II (Salomon & Perroteau, 1986). TGF beta, on the other hand, inhibits breast cancer cell proliferation *in vitro* (Knabbe *et al.*, 1987). Breast cancer cells in culture have also been shown to secrete a number of growth factors and to express cell surface receptors for some of these, such as EGFR. This has led to considerable speculation concerning the association of malignant transformation with the process of autocrine stimulation (Sporn & Roberts, 1985).

The 50 amino acid peptide, TGF alpha, is known to be derived from a 160 amino acid precursor molecule and is capable of binding to the EGF receptor (EGFR) and initiating cell division, as does EGF (Sporn & Roberts, 1985). It has been reported that TGF alpha synthesis is regulated via the oestrogen receptor (ER) (Lippman, 1985). To date there has only been a single report on the occurrence of TGF alpha transcripts in solid tumours (Derynck *et al.*, 1987). This showed that a TGF alpha mRNA of 4.5-4.8 kb was present in many different carcinomas, but only two breast cancers were studied. These workers failed to find TGF alpha transcripts in non-malignant adult tissue.

TGF beta is structurally and functionally distinct from TGF alpha. It is a 25 kDa protein composed of two identical subunits which have been found to be synthesised by a wide variety of normal and neoplastic cells (Derynck *et al.*, 1985). TGF beta mRNA has been detected in many solid tumours. One such study indicated that the TGF beta mRNA levels in tumour cells were generally not higher than in actively dividing normal cells, but these workers did find higher levels of TGF beta mRNA in the six carcinomas tested when compared with the adjacent normal tissue (Derynck *et al.*, 1987).

We have recently investigated the occurrence of growth factor transcripts in normal breast tissue, benign breast tumours and breast carcinomas and found that mRNA for TGF alpha and its receptor, EGFR, occurred together more commonly in oestrogen independent than in oestrogen dependent carcinomas. We also found that TGF beta mRNA was more abundant in carcinomas than in benign or normal breast tissue (Travers *et al.*, 1988).

We have now extended these observations to include a larger number of breast carcinomas and benign breast samples, and have examined the expression of transforming growth factor mRNAs in relation to survival and relapse-free survival in breast cancer patients.

### Materials and methods

### *Patients and samples*

Breast samples were collected at surgery and immediately frozen and stored in liquid nitrogen for 6-72 months. In this study we used 69 breast carcinomas (66 primary tumours and three biopsies of recurrent disease), 20 samples of benign breast disease (fibroadenoma, 12; mammary dysplasia, eight) and six samples of normal reduction mammoplasty specimens. In five cases the corresponding axillary lymph node metastasis was also obtained.

In all cases histological confirmation of diagnosis was obtained. Of the breast carcinomas, 54/69 (78%) were infiltrating ductal type, and 9/69 (13%) were lobular cancers. There were two mucinous and two medullary cancers, one tubular and one papillary cancer. Wherever possible, details on histological grade (Bloom & Richardson, 1957), T stage and nodal status at operation were obtained. In addition, frozen sections of 37 cancers were assessed for tumour cellularity on an arbitary scale, and the degree of lymphocytic infiltration was examined in 42 cases. The amount of tumour stroma (desmoplasia) was also recorded in 36 cases on an arbitrary scale as: 1, none seen; 2, slight; 3, slight to moderate; 4, moderate; 5, moderate to strong; 6, strong.

Patients were followed up to the end of January 1988. The time to relapse was defined as the period from primary surgery until recurrent disease. The site of recurrence was also noted. Overall survival was defined as the time from primary surgery to the end of the study. The mean duration of follow-up was 42.5 months with range 2-115 months.

### *cDNA probes*

The complementary DNA (cDNA) sequences encoding EGFR (Ullrich *et al.*, 1984), TGF alpha (Derynck *et al.*, 1984) and TGF beta1 (Derynck *et al.*, 1985) were excised

*Present address: Royal Marsden Hospital, Downs Road, Sutton, Surrey SM2 5PT, UK.
Correspondence: R.C. Coombes.
Received 7 June 1989; and in revised form 9 October 1989.

from plasmids and labelled with alpha-$^{32}$P-dCTP (Amersham, UK) by the random primer method (Feinberg & Vogelstein, 1983).

*Analysis of RNA*

Total cellular RNA was extracted from 0.5–1 g of frozen tissue as previously described (Chirgwin *et al.*, 1979). In 10 cases, polyadenylated (Poly(A)$^+$) mRNA was obtained by one passage through oligo (dT) cellulose (Aviv & Leder, 1972). For dot blot analysis serial dilutions of denatured total RNA were applied to Biodyne A membranes (Pall Filtration, Portsmouth, UK) using a Bio-Dot apparatus (Bio-Rad, UK), as previously described (Barrett-Lee *et al.*, 1987). Also, serial dilutions of the recombinant plasmid being studied and of non-homologous RNA were applied to each membrane in order to quantify the signal and to determine the extent of non-specific hybridisation.

For northern analysis (Thomas, 1980), 2.5 µg of poly (A)$^+$ mRNA per sample was resolved in a formaldehyde/agarose gel and blotted onto Biodyne A membrane. Denatured RNA/DNA markers were also run to enable sizing of hybridising bands.

Filters were pre-hybridised at 42°C for 6 h in 50% (v/v) deionised formamide, 0.1% sodium dodecyl sulphate (SDS), 5 × Denhardt's solution (1 × Denhardt's solution = 0.02% each of polyvinylpyrrolidone, bovine serum albumin and Ficoll), 5 mM EDTA, 0.75 M NaCl and 50 mM $NaH_2PO_4$ pH 8.3, and denatured sonicated salmon sperm DNA (250 µg ml$^{-1}$).

Filters were then hybridised overnight under the same conditions as for pre-hybridisation with the addition of 1–5 × 10$^6$ c.p.m. ml$^{-1}$ of denatured cDNA probe.

After hybridisation, filters were washed with three changes of 2 × SSC (20 × SSC = 3 M NaCl, 0.3 M trisodium citrate, pH7), 0.1% SDS at room temperature, and two changes of 0.1 × SSC, 0.1% SDS at 65°C. Autoradiography was carried out using Hyperfilm MP (Amersham, UK) with intensifying screens at −70°C, for 4–14 days.

Quantification of mRNA was carried out by comparison, with serial dilutions of the appropriate plasmid. A hybridisation intensity scale from 1 to 5 was derived corresponding to 7.8–125 pg of plasmid. Allowance was made for the proportion of recombinant insert to vector.

*Oestrogen receptor determination*

Measurement of ER was by a modification of the dextran-coated charcoal assay (McGuire & De La Garza, 1973). When samples were too small for this technique, ER was estimated by an immunocytochemical assay as previously described (McClelland *et al.*, 1986).

Using the biochemical assay, carcinomas with $\geqslant$ 10 fmol mg$^{-1}$ were considered ER positive, while with the immunocytochemical assay, tumours with > 10% staining were defined as ER positive.

*EGFR immunocytochemistry*

EGFR protein was visualised using the EGFR1 monoclonal antibody and the indirect immunoperoxidase technique (Waterfield *et al.*, 1982). Staining was assessed on a 0, +, + +, + + + score corresponding to absent, small numbers, moderate numbers and large numbers of tumour cells stained. Intensity of staining was not taken into account.

*Statistical analysis*

Analysis of data was by $\chi^2$ test with Yates' continuity correction. Survival tables were constructed and analysed by the log rank statistic. Correlations were by Spearman's rank correlation ($r_s$).

*DNA extraction*

DNA was extracted from tumours by saving this layer during the guanidinium/caesium chloride RNA extraction. Purification was performed using phenol chloroform (1:1) followed by precipitation with 0.1 volume of 3 M sodium acetate (pH 5.3) and 2.5 volumes of absolute alcohol. DNA samples were then digested with an excess of EcoR1 restriction enzyme.

*Southern analysis*

Electrophoresis and blotting on Biodyne A membranes was carried out as previously described (Southern, 1975). Hybridisation and autoradiography was as for northern analysis. Samples of normal human lymphocyte DNA were used as controls.

### Results

*TGF alpha*

TGF alpha mRNA was measured in 66 carcinomas by dot blot analysis; 28 (42%) contained detectable transcripts. Of 28 TGF alpha positive tumours 18 (64%) were also ER positive. Of the 38 carcinomas in which no detectable TGF alpha was found, 27 (71%) were ER positive. In general the level of TGF alpha mRNA was low, and thus only 'plus' or 'minus' scoring was given. Expression of TGF alpha was virtually confined to infiltrating ductal carcinomas with the exception of one medullary carcinoma; all eight lobular carcinomas were negative as was one mucinous and one tubular carcinoma.

In two patients the primary carcinoma and lymph node metastasis were both assayed. In one case, both tissues were negative for TGF alpha mRNA and in the other both were positive. The relationship between TGF alpha mRNA and tumour grade was examined in 49 carcinomas. Grades 1, 2 and 3 carcinomas were positive for TGF alpha in 3/9 (33%), 8/17 (47%) and 11/23 (48%) respectively, these differences not being significant ($\chi^2 = 0.6$, $P = 0.74$). In the cases where nodal status was known, 42% of node positive patients and 58% of node negative patients were TGF alpha positive. The relationship between TGF alpha expression and tumour stroma or desmoplasia was examined but no correlation was seen ($r_s = 0.01$, $P = 0.94$, $n = 36$).

Four breast carcinoma cell lines were also studied: MCF-7, T-47D, ZR-75 and MDA-MB-231. The first two exhibited TGF alpha expression.

Twelve fibroadenomas, seven biopsies from mammary dysplasia and six biopsies of histologically confirmed normal breast were studied. These contained significant levels of TGF alpha transcript in 4/12, 5/7 and 2/6 cases respectively. Levels were similar to those found in carcinomas.

Ten unselected breast carcinoma mRNAs were subjected to northern analysis. The eight positive for TGF alpha contained a 4.8 kb transcript but one cancer also contained a 2.2 kb species (Figure 1). No other mRNAs were seen.

To investigate whether high expression of TGF alpha mRNA was due to gene amplification or gene rearrangement, DNA was isolated from five carcinomas, three samples of mammary dysplasia and three samples of normal breast and analysed by Southern blotting. None of the carcinomas showed amplification compared to lymphocyte DNA but one case of mammary dysplasia showed a 50–100-fold amplification as did one sample of normal breast tissue (Figure 2). Figure 2 also shows the absence of a 19 kb band in a reduction mammoplasty specimen. The reason for this is not clear and is currently being investigated.

The relationship between the TGF alpha mRNA content of cancers and the patients' relapse-free survival and overall survival was also studied. The patient characteristics are shown in Table I. No association between TGF alpha mRNA and survival was demonstrated even though the TGF alpha negative group contained a higher proportion of T2 carcinomas (Table II).

**Figure 1** Northern analysis of TGF and EGFR transcripts. 2.5 μg of Poly(A)+ of each sample was resolved on formaldehyde/agarose gels, blotted onto nylon filters and hybridised to $^{32}$P-labelled cDNA probe as described in Materials and methods. The sizes of the hybridising bands in kilobases are shown on the right. **a**, TGF alpha: tracks 1–2 are Poly (A)+ from two human breast cancers. **b**, TGF beta: tracks a–c are Poly (A)+ from three human breast cancers. **c**, EGFR: tracks 1, 3 and 4 are Poly (A)+ from three human breast cancers, track 2 is Poly (A)+from breast cancer cell line MDA-MB-231.

*EGFR*

Sixty-four breast cancers were examined for EGFR mRNA by dot blot analysis. Thirty-five (55%) contained detectable transcripts for EGFR, the majority of these (85%) being only of intensity + and ++, with the remainder being +++ or more. Of the 35 EGFR positive carcinomas 18 (51%) were ER positive but 26/29 (90%) of the EGFR negative carcinomas were ER positive, this difference being highly significant ($P = 0.0001$). In contrast, all normal and benign breast tissues tested contained EGFR message.

No relationship was seen between EGFR mRNA level and tumour grade in 40 cases since grades 1, 2 and 3 carcinomas

**Figure 2** Southern analysis of the TGFa gene. Each 10 μg sample of DNA was digested with *ECoR*1 restriction enzyme and subjected to electrophoresis, southern blotting and hybridisation to $^{32}$P-labelled TGF alpha cDNA as described in Materials and methods. The sizes of the hybridising fragments in kilobases are shown on the right. Tracks A and F, reduction mammoplastry and mammary dysplasia respectively; tracks B–D, three infiltrating ductal breast cancers; track E, normal human lymphocyte DNA.

**Table I** Characteristics of patients studied

| | | *TGF alpha mRNA* | | *TGF beta mRNA* | |
|---|---|---|---|---|---|
| *Characteristic* | | *+ ve* | *– ve* | *Low + medium* | *High* |
| T-stage | T1 | 9 | 4 | 5 | 9 |
| | T2 | 10 | 20 | 14 | 15 |
| | T3 | 4 | 8 | 3 | 8 |
| | T4 | 2 | 2 | 3 | 1 |
| | N/K[a] | 3 | 4 | 5 | 2 |
| Nodal involvement | + ve | 10 | 15 | 13 | 11 |
| | – ve | 14 | 14 | 8 | 21 |
| | N/K | 4 | 9 | 9 | 3 |
| ER status | + ve | 18 | 27 | 20 | 25 |
| | – ve | 10 | 11 | 10 | 10 |
| EGFR mRNA | + ve | 18 | 17 | 12 | 20 |
| | – ve | 9 | 20 | 17 | 12 |
| | N/K | 1 | 1 | 1 | 3 |
| TGF beta mRNA[b] | Low | 1 | 3 | | |
| | Medium | 11 | 15 | | |
| | High | 16 | 20 | | |
| Total | | 28 | 38 | 30 | 35 |

[a]N/K, not known. [b]TGF beta mRNA: low = 0 or +, medium = ++ or +++, high = ++++ or +++++ on hybridisation intensity scale. This table demonstrates the characteristics of the patient's breast carcinomas and relates these to transforming growth factor mRNA content. Levels of growth factor mRNA determined as in Materials and methods.

were EGFR positive in 3/7 (43%), 6/14 (43%) and 12/19 (63%) respectively ($\chi^2 = 1.65$, $P = 0.44$). Only 1/9 lobular carcinomas was EGFR positive. All three biopsies of recurrent disease studied were EGFR positive.

We had information on nodal status in 42 cases but no correlation with EGFR mRNA was found ($\chi^2 = 0.25$, $P = 0.62$). In four patients, EGFR mRNA was measured in both primary tumour and nodal metastases and in all cases the levels in both samples were identical.

We also examined the breast carcinoma cell lines for the

**Table II** Growth factor expression and relationship to prognosis in breast cancer

| | | Survival | | | Relapse-free survival | | |
|---|---|---|---|---|---|---|---|
| *Growth factor/receptor* | *No.* | *Obs.*[a] | *Exp.* | *P value* | *Obs.* | *Exp.* | *P value* |
| TGF alpha mRNA | | | | | | | |
| + ve | 28 | 7 | 7.95 | 0.56 | 12 | 15.75 | |
| − ve | 38 | 7 | 6.05 | | 20 | 16.25 | 0.17 |
| TGF beta mRNA | | | | | | | |
| Low + medium | 30 | 8 | 7.98 | 0.99 | 19 | 14.73 | 0.11 |
| High | 35 | 5 | 5.02 | | 11 | 15.27 | |
| EGFR mRNA | | | | | | | |
| + ve | 35 | 6 | 6.31 | 0.86 | 16 | 16.72 | 0.79 |
| − ve | 29 | 8 | 7.69 | | 15 | 14.28 | |

[a]Obs. = observed number of events; exp. = expected number of events. *P* values determined from life tables using the log rank statistic.

presence of EGFR mRNA. MCF-7 and ZR-75 cells were weakly positive, MDA-MB-231 cells were intensely positive and T-47D cells were negative.

Three carcinomas, one fibroadenoma and one carcinoma cell line (MDA-MB-231) were subjected to northern analysis and, in all cases, three hybridising bands were seen, of 10, 6.4 and 4.8 kb (Figure 1).

To determine whether breast cancers and non-malignant breast tissue were expressing EGFR protein, an immunocytochemical study using a monoclonal antibody to EGFR was carried out. Frozen sections from a total of 24 carcinomas and three benign fibroadenomas were stained. In EGFR positive tumours, we observed specific staining of both the cytoplasm and the plasma membrane of tumour cells. There was considerable heterogeneity in the pattern of staining, such that within EGFR positive tumours some individual cells stained strongly, some weakly, and some had no staining. This pattern of heterogeneous staining was also seen in fibroadenomas and in normal breast ducts (Figure 3).

Immunocytochemical staining of EGFR was (as independently assessed by a pathologist) found to correlate well with the presence of EGFR mRNA within the tissues ($\chi^2 = 11.5$, $P = 0.0007$). Only 3/14 cancers expressing EGFR mRNA showed no antibody staining, while all 10 tumours with no EGFR message also stained negative for EGFR protein. All three of the fibroadenomas exhibited both EGFR mRNA and EGFR staining.

Both EGFR and its ligand, TGF alpha, were expressed simultaneously in 18/64 (28%) of carcinomas studied and eight of these were ER positive. Conversely, 20/22 carcinomas negative for both proteins were ER positive carcinomas ($P = 0.01$).

In 64 patients detailed clinical follow-up data were available. These demonstrated no correlation between EGFR mRNA levels in the primary tumour and the subsequent relapse rate and overall survival in breast cancer patients (Table II).

### *TGF beta*

A total of 65 carcinomas and 20 non-malignant breast tissues were examined for TGF beta mRNA and all contained this transcript. However, there was a clear difference in the level of TGF beta transcripts between benign and malignant samples since 35/65 (54%) of breast carcinomas had high levels (+ + + + or + + + + +) compared to only 3/20 (15%) benign samples ($\chi^2 = 8.65$, $P = 0.01$). This pattern of TGF beta expression was not a reflection of the varying degrees of cellularity of the breast cancers, since in 37 cancers there was no significant relationship between tumour cellularity and TGF beta mRNA ($r_s = 0.3$, $P = 0.07$).

These results contrasted with the pattern seen in breast cancer cell lines where only MCF-7 cells expressed high levels of TGF beta mRNA (+ + + +), while the other lines (T-47D, ZR-75 and MDA-MB-231) contained low or medium levels (+, + and + + respectively). Interestingly, normal human lymphocytes were found to contain high levels (+ + + + +) of TGF beta mRNA.

**Figure 3** EGFR immunocytochemistry of normal, benign and malignant breast tissue. Sections were incubated with the EGFR1 antibody (1:50 dilution) or control antibody and subjected to the horseradish peroxidase mouse-antihorseradish peroxidase procedure with visualistion by the diaminobenzidine method. Slides were counter stained with Harris haematoxylin. **a**, right, infiltrating ductal breast cancer exhibiting heterogeneous staining of the cell membranes of tumour cells (original magnification × 320); left, section from same cancer incubated with control antibody only (original magnification × 100). **b**, left, normal breast duct showing mainly cytoplasmic positive EGFR staining; right, benign fibroadenoma showing positive staining of the epithelial component. Note absence of stromal staining.

Since it has been suggested that expression of TGF beta may be influenced by oestrogen action, the expression of mRNA for this growth factor was examined in relation to ER-status in the 65 breast cancers. However, no such relationship was found in this series ($\chi^2 = 3.27$, $P = 0.19$). Also, there was no correlation between histological grade and levels of TGF beta message in the 41 breast carcinomas where this information was available ($\chi^2 = 3.39$, $P = 0.49$).

Data on nodal status at operation and TGF beta expression were available in 53 cases. When TGF beta expression was divided into low, medium and high, a clear relationship was demonstrated with tumours from node positive patients having significantly lower TGF beta mRNA levels compared to node negative patients ($P = 0.05$). When TGF beta expression was examined in relation to histological type, certain patterns emerged. Thus the nine lobular carcinomas were found to express significantly lower levels of TGF beta mRNA compared to 54 non-lobular cancers ($\chi^2 = 7.16$, $P = 0.028$).

Since high levels of TGF beta mRNA were found in normal human lymphocytes, the amount of peritumoral lymphocytic infiltration was assessed on frozen sections in 42 of the breast carcinomas. In eight cancers we observed no lymphocytes, in another two there was a very strong reaction, while the rest showed varying degrees of infiltration. In view of these findings, TGF beta expression was examined in relationship to lymphocytic infiltration in these 42 carcinomas, but no correlation was found ($r_s = 0.19$, $P = 0.22$).

Polyadenylated RNA from three carcinomas was analysed by northern hybridisation. In all cases a TGF beta transcript of 2.5 kb was found. No other bands were detected (Figure 1).

We examined the influence of tumour TGF beta mRNA levels on the survival of breast cancer patients. Although patients with higher levels of tumour TGF beta mRNA had slightly longer relapse-free survival, this difference was not significant. Similarly, overall survival was not seen to be related to TGF beta mRNA levels.

### Discussion

This study is principally concerned with determining the clinical significance of TGF synthesis by human breast cancers. Previous studies in breast cancer have been, in the main, limited to a few well-defined breast cancer cell lines (Lippmann *et al.*, 1986; Bronzert *et al.*, 1987; Peres *et al.*, 1987). Several groups have postulated a role for these factors in breast cancer cell proliferation based on *in vitro* studies. Very few data have been available on growth factor expression in non-neoplastic breast tissue.

In view of the fact that these peptides are locally produced and locally active it was felt important to study mRNAs encoding these substances, since these would more accurately reflect synthesis *in situ* rather than absorption or storage of peptide. Also there is evidence that, in some cases, growth factors produced by cancer cells are not secreted, but remain intracellular or cell-membrane associated (Stoscheck & King, 1986).

Our main observation is that these factors are capable of being synthesised by all breast tissues: normal, benign and malignant. TGF alpha mRNA synthesis does not appear to differ between malignant and non-malignant tissue but TGF beta mRNA is more abundant in breast cancer tissue when compared with non-neoplastic breast. Co-existence of both TGF alpha and its receptor, EGFR, was more often observed in ER negative breast cancers, and this may signify a role for this peptide in the oestrogen independent growth of these tumours. This has already been suggested by the finding (from both binding studies and immunocytochemistry) that EGFR is expressed predominantly in ER negative breast cancers (Fitzpatrick *et al.*, 1984; Sainsbury *et al.*, 1985).

None of the growth factors in this study demonstrated a relationship to either tumour cellularity or histological grade. This suggests that levels of growth factor mRNA are not simply related to cellular proliferation or mitotic rate. It has also been suggested that the desmoplastic reaction seen in many carcinomas may be due to the production, by tumour cells, of growth factors mitogenic to stromal cells (Ross *et al.*, 1986; Peres *et al.*, 1987). However, we have previously shown that no relationship exists between PDGF expression and stromal proliferation in human breast cancer biopsies (Travers *et al.*, 1988). In the present study the same lack of correlation with tumour desmoplasia was seen for transforming growth factor mRNA expression.

Transforming growth factor expression was found to be generally low or absent in infiltrating lobular breast cancers. Most of these tumours were also EGFR negative. Recently, differences in growth factor expression between different subtypes of human lung cancer have also been found (Soderdahl *et al.*, 1988). It is possible that this differential expression of growth factors may, in part, explain the distinct biological and clinical behaviour of histological subtypes of cancer.

We have also analysed the relationship of TGF mRNA synthesis to tumour characteristics and disease-free survival and overall survival of patients with breast cancer, but as yet no relationship has been found in these relatively small groups of patients. This does not mean that, with continued follow-up, and with larger numbers of tumours, such a relationship may not eventually emerge. In fact, the data already suggest that the possession of high levels of TGF beta mRNA within a tumour confers some protection from relapse, but this does not yet achieve statistical significance ($P = 0.11$). Furthermore, higher levels of TGF beta mRNA were shown to be weakly associated with the absence of nodal metastases at presentation.

The studies presented here do not throw any light on the site of synthesis of these peptides. It is likely that several or all cell types present within these biopsies are capable of TGF synthesis and it may be that information on the relative synthetic capacity of various cell types will help to clarify their role in tumour progression. To study this, we are currently investigating the localisation of mRNA for TGF alpha and TGF beta by *in situ* hybridisation.

We thank A. Ullrich for the gift of the cDNA probes. We also thank U. Berger for assessment of immunocytochemistry and histological tissue sections.

### References

AVIV, H. & LEDER, P. (1972). Purification of biologically active globin messenger RNA by chromatography on oligothymidylic acid-cellulose. *Proc. Natl Acad. Sci. USA*, **69**, 1408.

BARRETT-LEE, P.J., TRAVERS, M.T., MCCLELLAND, R.A., LUQMANI, Y. & COOMBES, R.C. (1987). Characterisation of estrogen receptor messenger RNA in human breast cancer. *Cancer Res.*, **47**, 6653.

BLOOM, H.J.G. & RICHARDSON, W.W. (1957). Histological grading and prognosis in breast cancer: a study of 1,409 cases, of which 359 have been followed for 15 years. *Br. J. Cancer*, **11**, 359.

BRONZERT, D.A., PANTAZIS, P., ANTONIADES, H.N. & 4 others (1987). Synthesis and secretion of platelet-derived growth factor by human breast cancer cell lines. *Proc. Natl Acad. Sci. USA*, **84**, 5763.

CHIRGWIN, S.M., PRZYBYLA, A.E., MACDONALD, R.J. & RUTTER, W.J. (1979). Isolation of biologically active ribonucleic acid from sources enriched in ribonuclease. *Biochemistry*, **18**, 5294.

DERYNCK, R., GOEDDEL, D.V., ULLRICH, A. & 4 others (1987). Synthesis of messenger RNAs for transforming growth factors alpha and beta and the epidermal growth factor receptor by human tumours. *Cancer Res.*, **47**, 702.

DERYNCK, R., JARRETT, J.A., CHEN, E.Y. & 6 others (1985). Human transforming growth factor-beta, complementary DNA sequence and expression in normal and transformed cells. *Nature*, **316**, 701.

DERYNCK, R., ROBERTS, A.B., WINKLER, M.E., CHEN, E.Y. & GOEDDEL, D.V. (1984). Human tranforming growth factor-alpha: precursor structure and expression in *E. coli*. *Cell*, **38**, 287.

FEINBERG, A.P. & VOGELSTEIN, B.A. (1983). A technique for radiolabelling DNA restriction endonuclease fragments to high specific activity. *Anal. Biochem.*, **132**, 6.

FITZPATRICK., S.L., BRIGHTWELL, J.S., WHITTLIFF, J.L., BARROWS, G.H. & SCHULTZ, G.S. (1984). Epidermal growth factor binding by breast tumour biopsies and relationship to estrogen receptor and progestin receptor. *Cancer Res.*, **44**, 3448.

KNABBE, C., LIPPMAN, M.E., WAKEFIELD, L.M. & 4 others (1987). Evidence that transforming growth factor-beta is a hormonally regulated negative growth factor in human breast cancer cells. *Cell*, **48**, 417.

LIPPMAN, M.E. (1987). Growth regulation of human breast cancer. *Am. Fed. Clin. Res.*, **28**, 375.

LIPPMAN, M.E., DICKSON, R.B., BATES, S. & 6 others (1986). Autocrine and paracrine growth regulation of human breast cancer. *Breast Cancer Res. Treat.*, **7**, 59.

McCELLAND, R.A., BERGER, V., MILLER, L.S., POWLES, T.J. & COOMBES, R.C. (1986). Immunocytochemical assay for estrogen receptor in patients with breast cancer: relationship to a biochemical assay and to outcome of therapy. *J. Clin. Oncol.*, **4**, 1171.

McGUIRE, W.L. & DE LA GARZA, M. (1973). Improved sensitivity in the measurement of estrogen receptor in human breast cancer. *J. Clin. Endocrinol. Metab.*, **37**, 986.

OSBORNE, C.K., HAMILTON, B., TITUS, G. & LIVINGSTONE, R.B. (1980). Epidermal growth factor stimulation of human breast cancer cells in culture. *Cancer Res.*, **40**, 2361.

PERES, R., BETZHOLTZ, B., WESTERMARK, B. & HELDIN, C.-H. (1987). Frequent expression of growth factors for mesenchymal cells in human mammary carcinoma cell lines. *Cancer Res.*, **47**, 3425.

ROSS, R., RAINES, E.W. & BOWEN-POPE, ? (1986). The biology of platelet-derived growth factor. *Cell*, **46**, 155.

SAINSBURY, J.R.C., FARNDON, J.R., HARRIS, A.L. & SHERBET, G.V. (1985). Epidermal growth factor receptors on human breast cancers. *Br. J. Surg.*, **72**, 186.

SALOMON, D.S. & PERROTEAU, I. (1986). Growth factors in cancer and their relationship to oncogenes. *Cancer Invest.*, **4**, 43.

SALOMON, D.S., ZWIEBEL, J.A., BANO, M., LASONCZY, I., FEHNEL, P. & KIDWELL, W.R. (1984). Presence of transforming growth factors in human breast cancer cells. *Cancer Res.*, **44**, 4069.

SOUTHERN, E.M. (1975). Detection of specific sequences among DNA fragments separated by gel electrophoresis. *J. Mol. Biol.*, **98**, 503.

SODERDAHL, D., BETSHOLTZ, C., JOHANSSON, A., NILSSON, K. & BERGH, J. (1988). Differential expression of platelet-derived growth factor and transforming growth factor genes in small- and non-small-cell human lung carcinoma lines. *Int. J. Cancer*, **41**, 636.

SPORN, M.B. & ROBERTS, A.B. (1985). Autocrine growth factors and cancer. *Nature*, **313**, 745.

STOSCHECK, C.M. & KING. L.E. (1986). Role of epidermal growth factor in carcinogenesis. *Cancer Res.*, **46**, 1030.

THOMAS, P.S. (1980). Hybridisation of denatured RNA and small DNA fragments transferred to nitrocellulose. *Proc. Natl Acad. Sci. USA*, **77**, 5201.

TRAVERS, M.T., BARRETT-LEE, P.J., BERGER, V. & 4 others (1988). Growth factor expression in normal, benign and malignant breast tissue. *Br. Med. J.*, **296**, 1621.

ULLRICH, A., COUSSENS, L., HAYFLICK, J.S. & 12 others (1984). Human epidermal growth factor receptor cDNA sequence and aberrent expression of the amplified gene in A431 epidermoid carcinoma cells. *Nature*, **309**, 418.

WATERFIELD, M.D., MAYES, E.L.V., STROOBANT, P. & 5 others (1982). A monoclonal antibody to the epidermal growth factor receptor. *J. Cell Biochem.*, **20**, 149.